# Injection of Tincture of Iodine into Suppurating Cavities

**Published:** 1849-02

**Authors:** H. M. Hard

**Affiliations:** Milwaukee, Wisconsin


ART. II.—*Injection of Tincture of Iodine into Suppurating Cavities.* By H. M. HARD, M. D., Milwaukee, Wisconsin.

The following cases, taken from my case book, illustrate in a striking manner, the utility of the above method of applying this valuable therapeutic agent.

July 4th. 1847.—G. J., a laboring man, applied to me with a disease in the axilla depriving him of the use of his arm. Three months previously he had received a penetrating wound in the axilla from a small stick, the wound had not healed—there had been a purulent discharge ever since. I found a tumor of the size of a large orange occupying the seat of the injury; it had a dark, dull red color—was very hard, and firmly adherent to the adjacent parts; several lymphatic glands, not connected with the tumor, were enlarged, and indurated. The center of this tumor was perforated by a suppurating sinus, into which I passed a probe to the depth of three inches.

The previous treatment had consisted of various unguents, poultices, &c., from which he had derived no benefit. After cleansing the sinus by injections of tepid water, I injected the Tinct. of Iodine, and directed the Unguentum Iodine to be applied to the tumor twice a day. I used no other iodine injections on alternate days, continuing the unguentum, and in ten days the sinus had healed from the bottom, and the tumor diminished to the size of a filbert, which soon after entirely disappeared.

April 17th, 1848.—J. P, commander of a brig; received a severe, lacerated and contused wonnd of the scalp from the falling of a spar upon his head. The wound commencing upon the apex of the forehead, near the mesian line, and at the border of the hairy scalp, traversed in a semilunar direction the right side of the head, terminating near the vertex, forming a somewhat serrated incision nearly five inches in extent. The scalp upon the concave side of the incision was completely reflected, forming a flap as large as the palmar surface of the hand, the hemorrhage had been rather free, but had nearly ceased upon my arrival. The cranium was apparently uninjured. There were no symptoms of concussion or compression. I removed all foreign bodies from the wound, and brought the edges of the integuments accurately together by tying portions of the hair across the line of incision at proper intervals, removed the remaining hair for some distance around, and applied a compress and bandage, directing the part to be kept wet in a spirituous lotion. Ordered a brisk cathartic to be given in six hours.

April 19, 4 o'clock, A. M.—was summoned in great haste—found him delirious, with intense febrile reaction, face flushed, eyes deeply injected, pulse 110, full and strong, intense pain in the head, scalp much tumefied over the right side of the head including the forehead and upper eyelids, considerable swelling about both ears, with pain and stiffness at the angles of the jaw. This febrile disturbance had been gradually supervening during the night and previous afternoon, till it had arrived at the degree of intensity above described.

*Treatment.*—Bled him to approaching syncope, applied cold to the head, stimulating applications to the feet, and gave a brisk purgative of calomel and jalap. The usual antiphlogistic measures were energetically employed, and the inflammatory symptoms were soon controlled. The wound now commenced suppurating, and a large portion of the surrounding scalp became oedematous, pitting upon pressure. The discharge of pus increased rapidly at each subsequent dressing, till, at the expiration of three days, over ℥ii was discharged *per diem*, and, indeed, such was the activity of the suppurative process, that serious apprehensions began to be entertained for the safety of the entire portion of reflected scalp. There being but slight cephalalgia, and no vascular excitement, I at once determined to use the injection of iodine to control, if possible, the rapidly increasing suppuration. Consequently, f ℥i, two thirds the ordinary strength, was thrown into the suppurating cavity. Fourteen hours afterward the wound was dressed, the discharge was found to have diminished nearly two thirds, and the color changed to a rusty tinge. The injection was repeated on alternate days. After the second injection there was very little discharge. The wound now healed rapidly, and in a very few days the reparative process was completed.

December, 1848.

